# Estrogen receptor, progesterone receptor, and Her 2 Neu positivity and its association with tumour characteristics and menopausal status in a breast cancer cohort from northern Pakistan

**DOI:** 10.3332/ecancer.2012.283

**Published:** 2012-12-11

**Authors:** Mohammad Faheem, Humera Mahmood, Mohammad Khurram, Uzma Qasim, Javaid Irfan

**Affiliations:** Oncology Department of Nuclear Medicine, Oncology & Radiotherapy Institute (NORI), Islamabad, Pakistan

**Keywords:** estrogen receptor, progesterone receptor, Her 2 Neu oncogene, postmenopausal, overall survival

## Abstract

**Objectives:**

To measure the frequency of estrogen receptor (ER), progesterone receptor (PR), and Her 2 Neu positivity and to study their association with tumour characteristics and menopausal status in a breast cancer cohort from Northern Pakistan.

**Methodology:**

Patients attending NORI with histopathologically proven diagnosis of invasive ductal carcinoma of the breast were included after providing informed consent. Age, menopausal status, and tumour size were recorded. The presence or absence of nodal involvement (including site and number), distant metastases, sites of metastases, skin involvement, chest wall involvement, recurrent disease, and bilateral breast cancer were noted along with ER, PR, and Her 2 Neu status. Mean ± standard deviations were calculated for continuous variables like age. Frequency and percentage were calculated for categorical variables like ER, PR, and Her 2 Neu status. Association of ER, PR, and Her 2 Neu status with menopausal status and tumour-related characteristics were sought employing *t *test, *x*^2 ^square test, and ANOVA wherever appropriate. *P* value >0.05 was considered significant.

**Results:**

There were 1226 patients included in the study. Mean patient age was 48.04 ± 11.97 years, and 743 (60.6%) patients were premenopausal. ER, PR, and Her 2 Neu were found positive in 763 (62.2%), 738 (60.1%), and 478 (38.9%) patients, respectively. Significant association (*P *< 0.05) was found between ER, PR positivity, and Her 2 Neu over expression with menopausal status, tumour size, involvement of skin, chest wall and lymph nodes and the presence of distant metastases. However, no significant association was detected between ER, PR, Her 2 Neu and recurrent disease.

**Conclusions:**

The frequency of expression of hormonal receptors in breast cancer patients from Northern Pakistan is the same as reported in the literature although overexpression of Her 2 Neu is a little higher in our population. There is an inverse relationship between hormonal receptors expression and Her 2 Neu expression. Postmenopausal women have a higher incidence of ER and PR positivity and Her 2 Neu negativity. ER and PR negativity and Her 2 Neu positivity are associated with more advanced disease and poor outcome.

## Introduction

Breast cancer is a major public health problem for women throughout the world. In the United States, breast cancer remains the most common cancer in women and the second most frequent cause of cancer death [1]. Breast cancer is the most frequently diagnosed cancer in Pakistani females [2]. Breast cancer has been found to constitute 33% of all cancers in females registered at the Nuclear Medicine, Oncology and Radiotherapy Institute (NORI) Islamabad [3]. There are a number of factors which determine the prognosis of disease and response to treatment. Prognostic factors are those which determine the outcome of disease in the absence of systemic treatment whereas predictive factors predict response to treatment [1]. Estrogen receptor (ER) and progesterone receptor (PR) expressions are the most important and useful predictive factors currently available. ER and PR are intracellular steroid hormone receptors which have received substantial attention since 1986. Measurable amounts of ER and PR are found in about 50–85% of patients with breast cancer. The frequency of positivity and the level of ER and PR increase with age, reaching their highest levels in postmenopausal women [4]. Men have a higher ER and PR expression as compared to female breast cancer patients without any association with histological prognostic markers [5]. 

Her 2 Neu gene amplification is another important prognostic and predictive factor for breast cancer. Approximately 20% of breast cancer patients have Her 2 Neu gene amplification which results in glycoprotein overexpression [1]. This oncogene is associated with tumour aggressiveness and chemoresistance [6]. Her 2 Neu is an important factor in predicting response to trastuzumab. Only Her 2 Neu positive breast cancer cases are responsive to this drug [7]. A number of studies have been performed internationally showing the frequency of ER, PR, and Her 2 Neu positivity in breast cancer and their prognostic and predictive significance. Generally, the frequency of receptor positivity is inversely correlated with Her 2 Neu [8–10]. ER, PR negativity, and Her 2 Neu positivity have been found to be associated with higher tumour grade, larger tumour size, higher degree of lymph node involvement, and aggressive histopathological type [11]. 

Contrary to Europe and America, in Pakistan more than 60% of breast cancer patients present at an advanced stage of the disease. This means the biological behaviour of breast cancer in Pakistani population is different. Very few studies have been done in the country to find out the clinicopathological significance of ER, PR, and Her 2 Neu. Oncology patients from Northern Pakistan which include the twin cities of Rawalpindi, Islamabad, upper Punjab, Khyber Pakhtunkhwa, Gilgit Baltistan, Kashmir, and Hazara attend NORI. This study was carried out to note the frequency of positivity of ER, PR, and Her 2 Neu and its association with menopausal status, and tumour-related characteristics in addition to recurrent disease and overall survival in breast cancer cohort from Northern Pakistan, registered at the NORI Hospital, Islamabad. 

## Methodology

This observational study was conducted in the Oncology Department of Nuclear Medicine, Oncology & Radiotherapy Institute (NORI), Islamabad, after review and approval by the Institutional Ethics Review Committee. Patients attending NORI with a histopathologically proven diagnosis of invasive ductal carcinoma of the breast were included consecutively from January 2006 to December 2009 after providing informed consent. The status of ER, PR, and Her 2 Neu was sought by immunohistochemistry, and the presence of Her 2 Neu oncogene was confirmed by the Fluorescent In situ Hybridization (FISH) technique in each patient. 

Age, menopausal status, tumour size, and the presence or absence of nodal involvement (including site and number), distant metastases, sites of metastases, skin involvement, chest wall involvement, recurrent disease, and bilateral breast cancer were noted along with ER, PR, and Her 2 Neu status on a specifically designed *pro forma*. Mean ± standard deviation was calculated for continuous variables like age. The frequency and percentage were calculated for categorical variables like ER, PR, and Her 2 Neu status. The association of ER, PR, and Her 2 Neu status with menopausal status and tumour-related characteristics was sought employing *t *test, *x*^2 ^square test, and ANOVA wherever appropriate. *P* value >0.05 was considered significant. 

## Results

There were 1,511 breast cancer patients that attended NORI during the study period. The ER, PR, and Her 2 Neu status of 285 of the patients could not be retrieved at the time of study compilation and these patients were excluded. The study thus comprised 1,226 patients. 

The mean patient age was 48.04 ± 11.97 years. Regarding menopausal status, 743 (60.6%) patients were premenopausal and the rest postmenopausal. There were 881 (71.9%) patients who had T3 tumours, i.e. >5 cm in size. Skin and chest wall involvement were observed in 460 (37.5%) and 197 (16.1%) patients, respectively. There were 990 (80.8%) patients who had lymph node positive disease. The frequency of the number of positive axillary lymph nodes is given in Table 1. Supraclavicular nodes were involved in 108 (8.8%) patients. There were 274 (22.3%) patients who presented with distant metastases. The most common site of distant metastases were bone (*n *= 202, 16.5%), followed by lung (*n *= 163, 13.3%), liver (*n *= 98, 8%), and brain (*n *= 27, 2.2%). One patient (0.1%) had omental deposits. Recurrent disease was noted in 237 (19.3%) patients. Fifty four (4.4%) patients had bilateral breast cancer. 

The frequency and percentage of patients with positive ER, PR, and Her 2 Neu are given in Table 2. Correlation of expression of ER, PR, and Her 2 Neu with menopausal status, tumour size, skin and chest wall involvement, nodal involvement, number of nodes involved, distant metastases, bilateral breast cancer, and recurrent disease is shown in Table 3. Significant association (*P *< 0.05) was detected between ER, PR status, and Her 2 Neu expression (Table 4). 

## Discussion

Prognostic and predictive factors are universally utilized in the management of breast cancer and can be used to stratify patients into two groups; those who are expected to derive the most benefit from adjuvant systemic therapy and those for whom the risks and costs of adjuvant therapy outweigh the expected benefit [12]. ER/PR status and Her 2 Neu gene amplification and/or overexpression are both prognostic and predictive. Generally, ER and PR positivity and Her 2 Neu gene negativity are associated with better prognosis and vice versa. 

The present study was carried out to determine the frequency of ER, PR, and Her 2 Neu positivity and correlation of expression of ER, PR, and Her 2 Neu with menopausal status and some pathological parameters, as well as their effect on recurrence rate and overall survival in breast cancer patients registered at NORI from Northern Pakistan. The results obtained were found to be more or less similar to those reported in the international literature with some exceptions. The mean age of the patients in the study conducted was 48.04 years, and 42.4% patients were in 4th decade of life. The youngest patient seen was 19 years old. This age distribution is significantly younger than what is currently seen in western and Arab countries [13, 14]. More studies are required to determine the predisposing factors in our patients. One possible explanation is that cousin marriages are very common in Pakistan and accordingly hereditary factors could play a role. Another factor could be the degree of obesity associated with a diet high in fat and carbohydrates and lack of physical activity. 

In the study conducted, ER and PR were found to be positive in 62.2 and 60.1% patients, respectively, whereas Her 2 Neu was positive in 38.9%. These figures are different from those reported in a series of 112 patients (ER positive 72.3% and Her 2 positive 11.6%) [15]. This study was conducted in a small number of patients, i.e. 112 and patient demographics were different. 

Correlation of ER, PR with Her 2 Neu showed inverse relationship in the study conducted. The percentage of patients with positive ER and PR was found to be low in Her 2 Neu positive patients (18.35%) and high in Her 2 Neu negative patients (43.31%). The same results have been reported by Farzami *et al *[16] who found that Her 2 Neu overexpression was higher in ER and PR negative cases than ER and PR positive patients. As reported in literature higher number of ER and PR positive patients were postmenopausal with *P *value <0.05, whereas overexpression of Her 2 Neu was found to be significantly high in premenopausal women (*P *< 0.05) in our study. A significantly high mean (*P *< 0.05) of ER and PR positively stained cells was observed in postmenopausal females compared to premenopausal women, and Her 2 Neu overexpression was seen only in premenopausal women in a study done by Hussein *et al *[17]. 

In our patients, a very strong association was also detected between ER, PR, and Her 2 Neu expression and menopausal status and pathological parameters including tumour size, involvement of skin, chest wall and lymph nodes and the presence of supraclavicular adenopathy and distant metastases (*P *< 0.05). ER, PR negativity, and Her 2 Neu positivity was found to be associated with worsening disease stage i.e. increasing tumour size, increased involvement of skin, chest wall, lymph nodes, and the presence of distant metastases. The same results have been reported in other studies [18–21]. However, such a significant association of PR and Her 2 Neu was not detected with the presence of bilateral breast cancer. In our study, the grade of the tumour and response to treatment could not be studied although the grade of the tumour is affected by receptor status and Her 2 Neu expression [22–25]. Moreover, significant association of ER, PR, and Her 2 Neu with recurrent disease could not be detected which is contrary to the international literature [18–21]. 

More extensive studies are required to assess response to treatment and survival benefit in both receptor positive and negative patients. To make national guidelines for treatment, the studies should cover the population from all over the country. We should be able to identify early stage ER, PR, and Her 2 positive breast cancer in order to achieve improved disease control and overall survival of Pakistani women with a breast cancer diagnosis. 

## Conclusions

The frequency of expression of hormonal receptors in breast cancer patients from Northern Pakistan is the same as reported in the literature although overexpression of Her 2 Neu is higher in our population. There is an inverse relationship between hormonal receptors expression and Her 2 Neu expression. Postmenopausal women have a higher incidence of ER and PR positivity and Her 2 Neu negativity. ER and PR negativity and Her 2 Neu positivity are associated with more advanced disease and poor outcome. 

## Figures and Tables

**Table 1:** Number of nodes involved.

No. of nodes involved	No of patients	Percent
1–4	320	26.1
5–9	400	32.6
10–20	206	16.8
>20	64	5.2
No nodes involved	236	19.2
Total	1226	100.0

**Table 2:** Frequency and percentage of ER, PR, & Her 2 Neu.

Status	No. and percentage of patients with ER status	No. and percentage of patients with PR status	No. and percentage of patients with Her 2 Neu expression
Positive	763 (62.2%)	738 (60.1%)	478 (38.9%)
Negative	463 (37.8%)	488 (39.8%)	748 (61.01%)

**Table 3:** Correlation of expression of ER, PR, & Her 2 Neu with different parameters.

Parameter		Status of ER (*P*value)		Status of PR (*P*value)		Status of Her 2 Neu (*P*value)	
	Positive	Negative	Positive	Negative	Positive	Negative
Menopause						
Present	345	138	326	157	160	321
Absent	418	325	412	331	318	427
	(0.0000001)		(0.00007381)		(0.0008864)	
Tumour size						
Up to 2 cm	22	12	21	13	16	21
>2–5 cm	219	92	202	109	109	196
>5 cm	522	359	515	366	353	531
	(0.002147)		(0.3383)		(0.0000001)	
Skin involvement						
Present	231	229	229	232	232	235
Absent	532	234	510	256	246	513
	(0.0000001)		(0.0000001)		(0.0000001)	
Chest wall involvement						
Present	88	109	77	120	91	114
Absent	675	354	661	366	387	634
	(0.0000001)		(0.0000001)		(0.1142)	
Nodal involvement						
Present	601	389	587	403	412	578
Absent	162	74	151	85	66	170
	(0.0382)		(0.3644)		(0.000004874)	
Number of nodes involved						
1–4	219	101	225	109	107	215
5–9	257	153	256	144	168	226
10–20	100	126	102	108	105	99
>20	30	34	63	59	53	100
Supraclavicular nodes						
Present	35	73	36	72	69	48
Absent	728	390	702	416	409	700
	(0.0000001)		(0.0000001)		(0.000004513)	
Distant metastases						
Present	119	155	118	156	150	132
Absent	644	308	620	332	328	616
	(0.0000001)		(0.0000001)		(0.0000001)	
Bilateral breast cancer						
Present	26	28	29	25	23	39
Absent	737	435	709	463	455	709
	(0.02897)		(0.5974)		(0.8598)	
Recurrence						
Present	136	101	130	107	104	141
Absent	626	362	608	381	374	607
	(0.1727)		(0.3640)		(0.3581)	

**Table 4:** Relationship of Her 2 Neu expression with ER and PR status.

Her 2 expression	Status of ER (*P*value)		Status of PR (*P*value)	
	Positive	Negative	Positive	Negative
Her 2 positive	225	253	227	251
Her 2 Negative	531	217	504	244
	(0.0000001)		(0.0000001)	
